# Comparative Retreatment Efficacy of Two Multi-File Systems with Different Access Cavity Designs: A Micro-Computed Tomography Study

**DOI:** 10.3390/medicina60121980

**Published:** 2024-12-02

**Authors:** Emine Odabaşı Tezer, Dilan Kırmızı, Mohamad Abduljalil, Fatma Basmacı, Arda Buyuksungur, Meltem Dartar Öztan

**Affiliations:** 1 Department of Endodontics, Faculty of Dentistry, Ankara University, Ankara 06560, Turkey; emiodabasi@gmail.com (E.O.T.); mdartar18@gmail.com (M.D.Ö.); 2 Department of Endodontics, Faculty of Dentistry, Near East University, Nicosia 99138, Mersin 10, Turkey; dilan.kirmizi@neu.edu.tr (D.K.); fatma.basmaci@neu.edu.tr (F.B.); 3 Department of Endodontics, Faculty of Dentistry, European University of Lefke, Lefke 99770, Mersin 10, Turkey; 4 Department of Basic Medical Sciences, Faculty of Dentistry, Ankara University, Ankara 06560, Turkey; abuyuksungur@ankara.edu.tr

**Keywords:** access cavity design, instrumentation, micro-CT, multi-file systems, retreatment

## Abstract

*Background and Objectives*: The access cavity design and instrumentation system could affect the remaining root canal obturation materials in root canal retreatment. This study aimed to evaluate the efficiency of two different multi-file systems in removing obturation materials with two different access cavities utilizing micro-CT scanning. *Materials and Methods*: Conservative access cavity (CAC) preparation was performed for 80 mandibular premolars. Then, root canal preparation was employed followed by obturation. The retreatment process began by dividing the samples into two separate groups: conservative (CAC) and traditional (TAC) access cavities. Subsequently, these groups were assigned to eight distinct subgroups (n = 10): Group 1; TAC and ProTaper retreatment system (PTR) with ProTaper Next (PTN); Group 2, TAC and PTR + ProTaper Ultimate (PTUL); Group 3, TAC and PTN; Group 4, TAC and PTUL; Group 5, CAC and PTR + PTN; Group 6, CAC and PTR + PTUL; Group 7, CAC and PTN; and Group 8, CAC and PTUL. The samples underwent micro-CT scans before and after the retreatment process, and the volume and percentages of remaining root canal filling material were calculated. Statistical analysis of the data was performed, and significance was determined at the 5% level. *Results*: The influence of the access cavity design (*p* = 0.500), the ProTaper system (*p* = 0.138), and the interaction of these variables (*p* = 0.513) was insignificant. However, group 3 (TAC and PTN) showed the highest percentage of remaining obturation materials at 29.53%, contrasting with group 6 (CAC and PTR + PTUL). *Conclusions*: Neither retreatment procedure succeeded in completely removing filling materials. Nevertheless, the impact of access cavity design, different multi-file systems, and their interaction on the remaining root canal obturation materials was deemed insignificant.

## 1. Introduction

Failure in root canal treatment is usually related to the presence of residual bacteria (persistent infection) or reinfection of an endodontically treated tooth (secondary infection) because of inadequate cleaning, disinfecting, shaping, and filling of the root canal system, leaving endodontic retreatment as the first therapeutic alternative [1]. The primary objective of nonsurgical retreatment is to ensure the thorough cleaning, reshaping, and refilling of the root canal system, ensuring the complete removal of filling materials, debris, and microorganisms [1]. Removal of root canal filling material is the most important factor in nonsurgical retreatment. Several techniques can be used to remove gutta-percha including the use of heat, solvents, mechanical instruments, and various combinations of these methods [2]. Various designs of nickel-titanium (NiTi) rotary systems have been developed to improve the effectiveness of gutta-percha removal. A widely used special NiTi retreatment system is the ProTaper Universal retreatment system (PTR; Dentsply Maillefer, Ballaigues, Switzerland), which functions through continuous rotary motion [2]. The ProTaper Next (PTN) system (Dentsply Maillefer, Ballaigues, Switzerland) is a multi-file system constructed from M-Wire alloy. Although primarily designed for endodontic treatment, numerous previous studies have demonstrated its effectiveness in retreatment procedures [3,4,5].

The ProTaper Ultimate (PTUL) rotary system, developed by Dentsply Sirona Endodontics, represents the newest evolution of the ProTaper family. Distinguished by their innovative mechanical properties, these files aim to achieve an optimal balance between flexibility and strength [6]. It was also reported that the PT Ultimate system demonstrated reduced torsional strength and increased flexibility compared to other systems. Nevertheless, due to limited information available on this new system, additional research is necessary to inform clinical guidelines [7]. While the motor-driven NiTi instrument design is now favorable for the coronal root canal filling material removal, to date, no study has demonstrated removal of whole obturation materials during root canal retreatment, regardless of the technique or instruments used [2,8].

The access cavity design plays a crucial role in root canal treatment. In the traditional access cavity (TAC) design, the removal of the pulp chamber roof allows for straight and direct access to the canals, enhancing the efficiency of instrumentation [9]. Nonetheless, this approach entails significant removal of tooth structure, consequently diminishing the tooth’s fracture resistance [10]. As a result, a conservative access cavity (CAC) design has been devised as an alternative to the conventional access cavity approach [11].

In a study based on literature research, the impact of access cavity design on a total of 11 different treatment parameters such as retreatment, uninstrumented areas, and ability to locate the canal was evaluated according to different access cavity designs [12]. Only two studies in the searched literature evaluated the impact of the endodontic design of the access cavity on retreatment procedures [8,13]. The results showed that with minimally invasive approaches, more gutta-percha remained and longer time was required to eliminate the gutta-percha from the canal. One study suggested that the selection of rotary files, especially those with minimally invasive designs, might contribute to minimizing gutta-percha remaining within the canal [12]. Among these, only two studies by Niemi et al. [8], and Fatima et al. [13] compared TAC and CAC.

Micro-computed tomography evaluation has been widely used to evaluate the amount of residual root filling material in recent studies [1,4,5]. It is accepted as the gold standard because it is a reproducible and non-destructive method that provides a detailed evaluation of the three-dimensional morphological features of the observed object and also provides quantitative data [5]. It has proven successful in assessing the quantity of filling material within root canals, determining the amount of dentin removed during initial preparation, and evaluating the remaining filling material and dentin post-retreatment [1,4,5].

In the literature, there are few studies investigating the efficacy of different access cavity designs on retreatment. In addition, there are no studies investigating the efficacy of the PTUL system on retreatment or comparing this efficacy with different techniques. Hence, the purpose of this experiment was to evaluate the efficiency of PTUL and PTN systems in removing obturation materials during retreatment of single-rooted mandibular premolars with two different access cavities, utilizing micro-CT scanning. The hypothesis of the present study is that root canal filling material removal will be more efficient when utilizing a traditional access cavity design compared to a conservative access cavity design.

## 2. Materials and Methods

**Figure 1:**
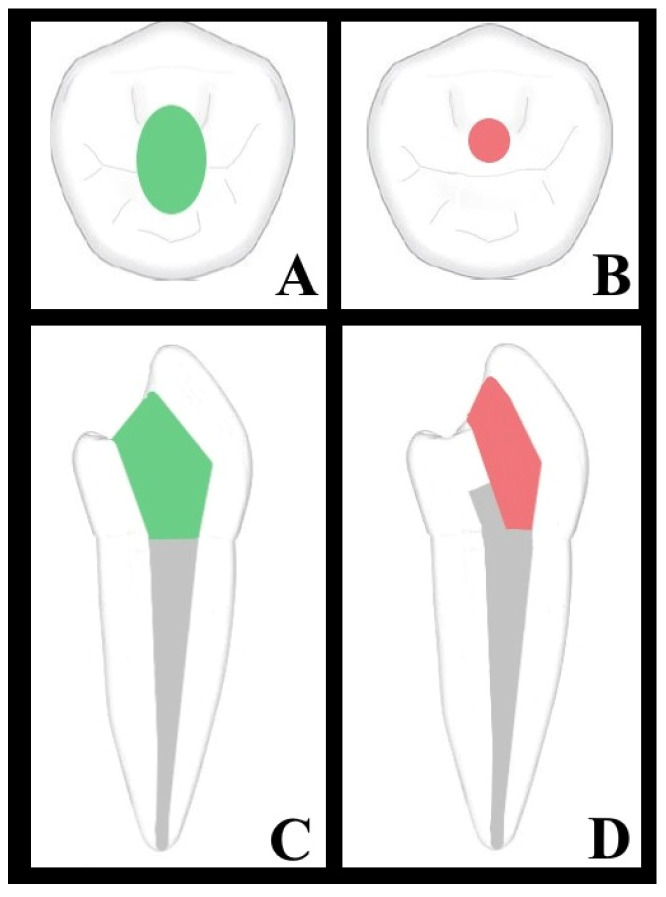
Diagrammatic reconstructions of mandibular premolars illustrate traditional and conservative access cavity designs, with the traditional cavity segmented in green (occlusal view, (**A**); sagittal view, (**C**)) and the conservative cavity in red (occlusal view, (**B**); sagittal view, (**D**)).

Approval for the research protocol was granted by the university’s ethical committee under reference number (36290600/23/2023). Eighty extracted mandibular premolars from human subjects were collected from the faculty of the maxillofacial surgery department, and radiographs were taken from the proximal section of the teeth. The teeth were selected based on criteria including intact crowns, completed root development, possessing a single root and canal, measuring 21–24 mm in length, and having wide canals from the buccal to lingual direction. A single practitioner conducted all of the endodontic procedures. To reduce bias in our study, the operator responsible for performing the retreatment procedures (E.O.T.) was blinded to the study’s objectives and anticipated outcomes. This blinding was implemented to ensure that the procedures were conducted without any influence from the study’s aims, thereby enhancing the reliability of our findings. The operator involved in the study is well-prepared and highly skilled in the techniques and protocols being investigated. With over 10 years of experience in the field of endodontics, the operator is also familiar with equipment such as rotary instrumentation systems. A high-speed mosquito 392 bur (Spring Health Diamonds, St. Louis Park, MO, USA) with water spray was used for conservative access preparation, and a new bur was employed for each specimen to ensure precision and consistency. The teeth were accessed 1 mm buccal to the central fossa, and cavities were extended apically, preserving part of the chamber roof and lingual shelf (Figure 1). Following this, the working length was determined by withdrawing 0.5 mm from the point where the tip of a ISO #10 K-type file (Dentsply Sirona, York, PA, USA) was observed at the apical foramen. A confirmatory radiograph was obtained to verify the accurate determination of the working length, and the corresponding measurements were documented for each specimen. Exclusion criteria eliminated teeth with previous endodontic treatment, significant restorations, resorption, calcifications, anatomical anomalies, or visible cracks. However, before canal preparation, only root canals where a #15–20 K-type file could reach the working length, but a #25 K-type file could not, were included in our study to standardize canal width.

### 2.1. Initial Root Canal Treatment

Subsequently, the root canals of each specimen were prepared using the ProTaper Universal system up to size F2 (Dentsply Tulsa Dental Specialties). The irrigation protocol employed in the canals was as follows: after obtaining each file, the irrigation process involved the use of 2 mL of 5.25% NaOCl, followed by 5 mL of 17% EDTA for 3 min, and finally irrigated with 5 mL of 5.25% NaOCl. A 31-G double side-port needle (NaviTip; Ultradent, South Jordan, UT, USA) was positioned 1 mm above the established WL and moved in a 1–2 mm motion in an in-and-out direction. Following the completion of all procedures, the canals were rinsed with saline and dried using paper points (Dentsply DeTrey, Konstanz, Germany). All specimens were filled utilizing the ProTaper Universal system F2 (25/.04) gutta percha master cones (Dentsply Tulsa Dental Specialties) and AH Plus root canal sealer (Dentsply De Trey GmbH, Konstanz, Germany) adapted to the canal walls using the lateral compaction technique.

Specimens demonstrating a filling without any gaps extending from the canal orifice to the root apex in the radiographs taken from the proximal aspect of the samples were considered eligible for inclusion in the study. Samples displaying voids in the root canal filling were replaced.

The access cavities of all samples were sealed with temporary filling material (Cavit G, 3M ESPE, Neuss, Germany) and then stored in a 37 °C environment with 100% humidity for 30 days to allow complete hardening of the root canal sealer. After the 30-day period, the samples underwent the first micro-CT scan, and the volume and percentage of root canal filling material for each sample were calculated.

### 2.2. Retreatment Process

For the retreatment procedure, the teeth were randomly assigned to two groups characterized by access cavity designs: the conservative access cavity group and the traditional access cavity group. In conservative group, temporary filling material was exclusively removed from 40 samples. The remaining 40 samples underwent enlargement utilizing the traditional access cavity method with a high-speed diamond round bur with water-cooling. This approach facilitated direct access into the filling material and complete removal of the pulp chamber and pulp horns (Figure 1).

The process of removing filling material from the root canals began by dividing the samples into two separate groups, as previously mentioned, depending on different access cavity designs. Subsequently, these groups were randomly assigned to four distinct subgroups according to the retreatment procedure technique (n = 10).

Group 1 (TAC and PTR + PTN): The access cavities of the samples belonging to this group were expanded to ensure direct entry to the canal filling material and pulp chamber, while also eliminating any remnants of pulp horns using a diamond bur under high-speed water cooling as a traditional access cavity design. A new bur was used for each sample tooth. The major portion of the obturation material was excised utilizing ProTaper Universal Retreatment instruments (manufactured by Dentsply Tulsa Dental) set to predetermined lengths of D1 (30/0.09), D2 (25/0.08), and D3 (20/0.07), operating at a 600 rpm. Subsequent to this, reinstrumentation with PTN was conducted according to the following protocol: X1 (017 0.04) and X2 (025 0.06) operating at a 300 rpm.

Group 2 (TAC and PTR + PTUL): The access cavities of the samples belonging to this group were expanded to ensure direct entry to the canal filling material and pulp chamber, while also eliminating any remnants of pulp horns using a diamond bur under high-speed water cooling as a traditional access cavity design. The obturation material was eliminated utilizing ProTaper Universal Retreatment instruments at D1, D2, and D3. Furthermore, in addition to this procedure, reinstrumentation performing PTUL was conducted according to the following protocol: Slider (016.002v), Shaper (020.004v), F1 (020.007v), and F2 (025.008v) operating at a 400 rpm.

Group 3 (TAC and PTN): The access cavities of the samples belonging to this group used a conventional access cavity design. Obturation material was removed using PTN instruments in the order described for the previous groups.

Group 4 (TAC and PTUL): The access cavities of the samples belonging to this group used a conventional access cavity (TAC) design. The obturation material was excised utilizing up to F2 with the previously mentioned PTUL application.

Group 5 (CAC and PTR + PTN): In this group, canal filling material was obtained by removing only the temporary filling without disrupting the previously conventionally prepared access cavity design. As noted in the previously explained procedure, the canal filling material was removed using PTR files then PTN files.

Group 6 (CAC and PTR + PTUL): CAC cavity design was also used in this group. The canal filling material was removed according to the previously reported technique by first using PTN files and then PTUL files.

Group 7 (CAC and PTN): The CAC cavity design was used within this group. The canal filling material was removed only using PTN files.

Group 8 (CAC and PTUL): In this group, the CAC cavity design was again used; the canal filling material was removed only with PTUL files.

Following each use of an instrument, the canals were flushed with 5.25% NaOCl. Retreatment was deemed finished when instruments were void of visible obturation material upon removal. Each specimen received a new batch of instruments for the procedure. Throughout the procedure, all specimens received irrigation with 5.25% NaOCl between instrumentations. Subsequently, final irrigation was performed on all specimens with 5 mL of 17% EDTA (Dentsply Tulsa Dental Specialties), followed by 5 mL of 5.25% NaOCl with the same irrigation protocol mentioned in the initial root canal irrigation.

Following the procedure, the pulp chamber was sealed with temporary filling material to avoid the risk of potential debris entering the root canal system. Subsequently, all specimens were prepared for another micro-CT scan to assess the volume and percentage of residual root canal filling materials within the root canal.

### 2.3. Micro-CT Scanning

Each specimen was imaged using a high-resolution micro-CT scanner (Bruker SkyScan 1275, Kontich, Belgium). The settings were configured to 125 mA and 80 kVp with a 1 mm aluminum filter, and the rotation steps were set to 0.2 degrees with a pixel size of 26.5 μm. Flat field correction was performed prior to scanning. Each sample completed a full rotation within an integration time of 30 min. The contrast limits were consistently maintained between 0.00 and 0.30 for all samples.

### 2.4. Imaging Analysis

Visualizations and quantitative analyses of the specimens were performed using CTAn software (version 1.23.0.2, Bruker SkyScan) and NRecon software (version 1.7.4.6, Bruker SkyScan) with a modified algorithm as described by Feldkamp et al. [14]. Two-dimensional axial images of 1000 × 1000 pixels were acquired, with beam artifact corrections set to 38% after smoothing 3, and ring artifact corrections set to 7 for the reconstruction parameters. NRecon software facilitated the reconstruction of the images acquired from the scanner, enabling the visualization of 2D slices of the specimens. CTAn software was utilized to delineate regions of interest (ROIs) encompassing the entirety of each specimen and to conduct post-reconstruction sample analysis. Models were created using CTAn software and visualized with CTVol version 2.3.2.0.

### 2.5. Statistical Analysis

The study’s sample size adequacy was examined to ensure robust statistical power for all analyses. Each group had a minimum sample size of 10 teeth determined through power analysis (utilizing G*Power 3.1.9.7 software from Heinrich Heine University, Dusseldorf, Germany) with a power of 80% and a type I error rate of 0.05.

Statistical analysis utilized the software program IBM SPSS Statistics (version 26; IBM Corp., Armonk, NY, USA). The normality assumption of the data was verified using the Shapiro–Wilk test. As the data exhibited a normal distribution (*p* > 0.05), parametric test was employed. A two-way ANOVA was conducted to assess the impacts of various access cavity designs and ProTaper systems on root canal retreatment. Significance was determined at the 5% level.

## 3. Results

**Figure 2:**
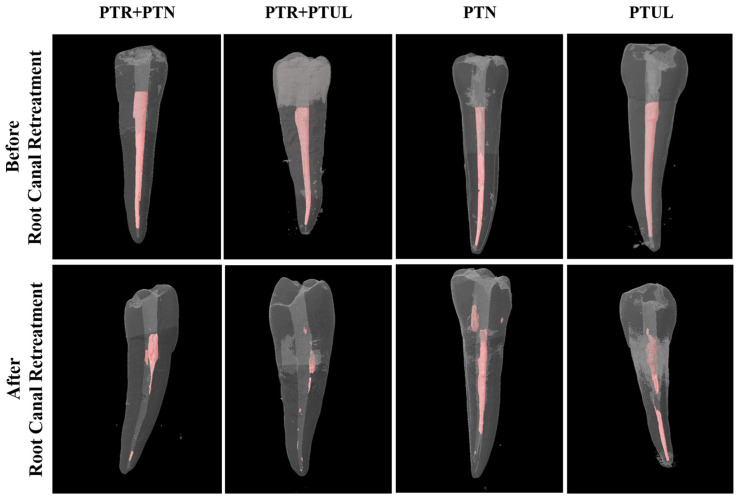
Examples of micro-CT images before and after root canal retreatment for each group with traditional access cavities.

**Figure 3:**
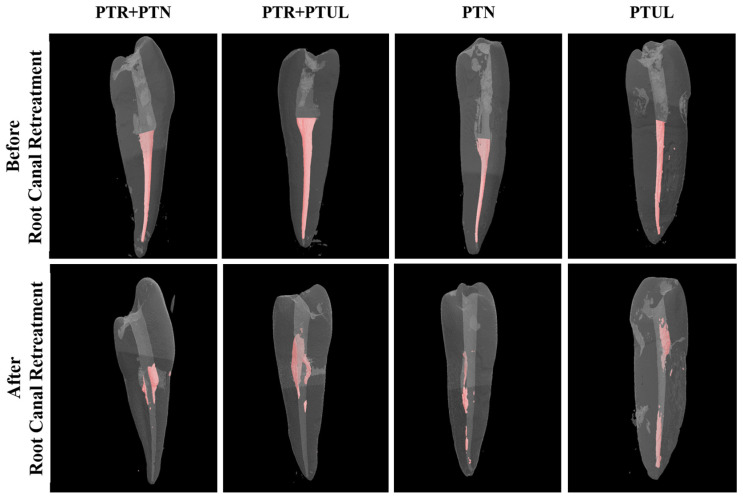
Examples of micro-CT images before and after root canal retreatment for each group with conservative access cavities.

The mean percentages of remaining root canal materials along with their respective standard deviations are depicted in Table 1. Examples of micro-CT images before and after root canal retreatment are shown in Figure 2 and Figure 3.

According to the results of the two-way ANOVA, the influence of access cavity design (*p* = 0.500), the ProTaper system (*p* = 0.138), and the interaction of these variables (*p* = 0.513) on the remaining root canal obturation materials was found to be insignificant. Given the lack of significant effects identified using the two-way ANOVA test, no multiple comparisons were conducted between the groups in the current study.

However, it is worth noting that group 3 (TAC and PTN) showed the highest percentage of remaining obturation materials at 29.53%, contrasting with group 6 (CAC and PTR + PTUL), which had the lowest value at 12.11%. Furthermore, despite the absence of statistical significance between the groups, both group 1 (TAC and PTR + PTN) and group 2 (TAC and PTR + PTUL) displayed similar percentages, with values of 16.92% and 16.97% respectively. However, the effectiveness of PTUL and PTN systems in removing root canal filling materials was statistically similar regardless of the access cavity design.

## 4. Discussion

Various techniques have been employed to measure the elimination of residual obturation material from root canals following endodontic retreatment [15,16]. Recently, there has been a rise in the utilization of advanced micro-CT scanning techniques, which have become favored for the ability to generate high-resolution 3D volumetric data. This non-invasive method is well-suited for analyzing, quantifying, and visualizing results in endodontic retreatment scenarios [17]. It has proven successful in assessing the quantity of filling material within root canals, determining the amount of dentin removed during initial preparation, and evaluating the remaining filling material and dentin post-retreatment [18]. Certain limitations of this technique stem from methodological factors, including the necessity for adequate training and proficiency in hardware and software operation. Achieving successful imaging of high-density tissues with micro-CT entails the ability to adjust x-ray energy appropriately by selecting the correct voltage/filter combination and applying suitable processing techniques to the original grayscale images to reduce noise, such as beam hardening correction [19]. In the present study, scanning was conducted at an optimal resolution of 26.5 μm, spanning 360 degrees, using a high X-ray voltage, a robust 1 mm aluminum filter, and three-frame averaging at each rotation step. These parameters facilitated the analysis of dentin adjacent to the filling material while minimizing artifacts [18].

The literature indicates that no single instrument or method can entirely eliminate root canal filling materials during nonsurgical root canal retreatment [3,4,20], potentially leading to treatment failures due to residual microorganisms in the canal system [21]. Consequently, novel instruments and additional protocols have been suggested to address this issue, especially when dealing with oval-shaped root canals where filling material tends to accumulate in polar areas, impeding its complete removal [22]. In this study, we utilized the PTN alongside the innovative PTUL files for retreatment procedures. To the best of our understanding, previous studies did not employ the ProTaper Ultimate system.

Creating an access cavity is the initial process in root canal treatment, crucial for treating pulpal and periapical infections. It is challenging but essential for successful treatment, enabling removal of obstructions, locating canal openings, and cleaning the root canal. Opening inappropriate access cavity may cause errors and endodontic therapy failure. Therefore, meticulous design and preparation are vital for quality treatment and preventing complications [23]. This study aimed to assess how two variant endodontic access cavity shapes affect the removal of obturation materials. To our knowledge, limited studies have examined the impact of different access cavity shapes on the removal of root canal obturation materials [8,13].

However, according to the findings of this paper, the influence of endodontic access cavity design, the ProTaper system, and their interaction on the remaining root canal obturation materials was insignificant. Thus, the hypothesis was rejected. Despite the shape of the access cavity, there was no significant difference between PTN and PTUL in their ability to remove filling materials. This aligns with prior studies’ conclusions, which also found no significant difference in retreatment efficacy between PTN and other NiTi systems [3,24]. On the contrary, Tantiwanichpun and Kulvitit [25] noted a significant difference among the PTNc and VR groups across the entire canal.

ProTaper Ultimate represents a progression from the well-received ProTaper Universal and ProTaper Gold systems, maintaining the same underlying philosophy and procedural approach as its predecessors. However, it offers enhanced advantages such as high flexibility, high cyclic fatigue resistance, and ease of use [6]. The instruments of the ProTaper Universal and Ultimate systems featured symmetrical blades devoid of flat sides or radial lands, boasting parallel surface finishing and an approximately similar nickel/titanium ratio [7], which presents a similar influence when used in the removal of root canal obturation materials. While a PTN file utilizing asymmetric motion delineates a broader motion envelope in contrast to a file of similar dimensions with a centralized mass and rotation axis, the current study revealed that the difference between the PTUL and PTN systems was statistically insignificant. This lack of distinction could be attributed to the superior quality features of the Ultimate file, including rotation with a higher angle, lower maximum torque, and minimized bending load with higher flexibility [7].

Regardless of the instrument used or access cavity design, patency was restored successfully in all teeth during retreatment. Previous papers have evaluated the influence of different NiTi systems on removing the root canal obturation materials, with or without the use of ProTaper Universal retreatment instruments [8,26]. Consequently, the present research was designed to assess the impact of PTN and PTUL systems in removing obturation materials with and without the incorporation of ProTaper Universal retreatment instruments. However, while the CAC with PTR + PTUL group exhibited the lowest value at 12.11%, the use of ProTaper Universal retreatment files as the initial step resulted in statistically similar outcomes compared to the groups where it was not employed.

CACs point to implementing partial removing of the pulp chamber roof with saving of pulp horns. The walls of the access cavity are occlusally beveled and slightly convergent. The promoted durability to fracture has been certainly remarkable in mandibular premolar and molar teeth, which appears to be less severe in CACs, particularly regarding cuspal chipping [27,28]. While many acknowledge that CACs might promote the resistance of teeth, with endodontic treatment, concerns have been raised regarding the ability to effectively shape, clean, and fill them. Additionally, the risk of procedural mistakes is heightened due to diminutive direct visibility. Some studies propose that using rotary instrumentation with higher fatigue resistance could mitigate this hazard [27]. The increased probability of overlooking root canal anatomy is another issue, especially the second mesiobuccal canal of maxillary molars. This can be difficult to visualize on diagnostic x-rays such as CBCT images [28]. In this study, the design of the CAC access outline adhered to the model suggested by Krishan et al. [28]. However, standardizing conservative access cavity (CAC) preparation is challenging due to variations in tooth anatomy, operator skill, instrument wear, and the need to balance tooth preservation with canal access, all of which introduce inconsistencies across samples, which was a limitation of our research. Due to the narrow dimensions of the conservative access cavity, the removal of obturation materials from the canal may become more challenging, potentially leading to a higher risk of procedural errors compared to traditional access cavities. However, our findings showed that the percentages of remaining root canal filling were statistically comparable between both access cavity designs. This suggests that using a CAC may offer more advantages, as it preserves dental tissue without compromising the removal of root filling materials, compared to the TAC, according to our study results.

There is a scarcity of literature concerning the evaluation of different access cavity shapes in retreatment. However, consistent with our findings, a study by Xia et al. [29] reported that the differences among CAC and TAC groups are insignificant regarding deviation of the central point in mandibular premolar teeth and increased sectional area. Additionally, they noted that the value of residual pulp tissue in isthmus and root canals in maxillary first molars was similar in both shapes. These observations may be attributed to the selection of premolar teeth with a single canal, which reduces the drawbacks of CAC and suggests its safety and efficacy in single-rooted teeth. Consequently, further investigations are warranted to assess the effect of CAC on other types of teeth.

On the contrary, prior studies have highlighted significant differences between TAC and CAC regarding fracture resistance and root canal preparation performance [28,30]. Although Neimi et al. [8] noted a significant overall difference between CAC and TAC designs, with CAC leading to more residual root canal obturation materials on canal surface, they found that the differences among the combinations of TAC-VB, CAC-TS, and TAC-TS were not significant. However, the incorporation of CAC and VB systems showed significantly higher values of residual filling materials on the canal surface in comparison to the other groups. Also, Fatima et al. [13] suggest a noteworthy distinction between CAC and TAC access designs. Specifically, they observed a substantial difference, indicating that CAC resulted in a greater quantity of filling material remaining on the root canal surface. This insight underscores the potential impact of access design on the efficacy of obturation procedures in endodontic treatments.

The duration of the retreatment procedure was not assessed in the present research due to limitations in the methodology employed. Also, the present study investigation was performed on intact noncarious premolars. However, clinically, most teeth that require root canal therapy are structurally compromised with caries; thus, CAC preparation design may not be practical in most of the cases. Standardizing conservative access cavity (CAC) preparation is challenging due to variations in tooth anatomy, operator skill, instrument wear, and the need to balance tooth preservation with canal access, all of which introduce inconsistencies across samples. In addition to the challenges associated with standardizing root canal anatomy in this study. Future studies should address this gap and explore the impact of new instrument types on the removal of filling materials in access cavities of various shapes on different types of teeth.

## 5. Conclusions

Despite the constraints of this in vitro study, neither retreatment procedure succeeded in completely removing filling materials from the root canals. Nevertheless, the impact of access cavity design, the ProTaper system, and their interaction on the remaining root canal obturation materials was deemed insignificant. However, our findings showed that the percentages of remaining root canal filling were statistically comparable between both access cavity designs. This suggests that using a CAC may offer more advantages, as it preserves dental tissue without compromising the removal of root filling materials, compared to the TAC, according to our study results.

## Figures and Tables

**Table 1:** The mean percentages of remaining root canal materials along with their respective standard deviations and two-Way ANOVA results.

Group	N	Mean	Std. Deviation			
				Two-Way ANOVA		
TAC and PTR + PTN	10	16.9264	4.94	Access Cavity Design	Instrument System	Access Cavity Design and Instrument System Interactions
TAC and PTR + PTUL	10	16.9777	5.77			
TAC and PTN	10	29.5360	7.93			
TAC and PTUL	10	25.6216	8.6			
CAC and PTR + PTN	10	21.1788	6.24			
CAC and PTR + PTUL	10	12.1120	3.87	0.500 *	0.138 *	0.513 *
CAC and PTN	10	18.6927	5.005			
CAC and PTUL	10	26.7768	4.55			

* *p* values ≥ 0.05 showed insignificant differences regardless the access cavity design and instrument system.

## Data Availability

Dataset available on request from the authors.
